# A pharmacist's role in increasing access to menstrual products: an education and advocacy approach

**DOI:** 10.3389/frph.2024.1364698

**Published:** 2024-05-17

**Authors:** Kristal Potter, Jessica Beal-Stahl

**Affiliations:** ^1^ Department of Clinical and Administrative Sciences, Larkin University College of Pharmacy, Miami, FL, United States; ^2^ Department of Clinical Research, Sports Pharmacy Network, Naples, FL, United States

**Keywords:** menstruation, menstrual cycle, period product insecurity, period poverty, patient education, tampon tax, toxic shock syndrome (TSS), pharmacist

## Abstract

Individuals who menstruate grapple with diverse challenges in menstrual and reproductive health. This includes financial burdens, societal stigmas, and negative mental and physical health implications. Period poverty, marked by insufficient access to menstrual products, education, and sanitation, remains a prevalent and poorly addressed issue. Alarming statistics highlight the extent of this problem and shed light on the staggering number of individuals lacking access to essential menstrual products. The discourse extends to the safety and accessibility of a diverse array of menstrual products. A comprehensive comparison of the cost of available period products was conducted using data obtained from various retail websites. The often-overlooked potential indirect expenses and profound impacts on quality of life were also discussed. Amidst other public health initiatives, pharmacists have emerged as pivotal advocates and educators. Pharmacists are poised to drive initiatives that increase access to menstrual products through public health education and advocacy. By providing education on different menstrual product options, pharmacists can empower individuals to make informed decisions based on their needs. This perspective illuminates the complex impacts of menstruation on individuals and proposes that pharmacists can play a role in overcoming barriers to access. The proposed strategies, rooted in education, research, and advocacy, pave the way for enhancing access, reducing stigma, and elevating the quality of life for those navigating the intricate complexities of menstruation.

## 1 Introduction

When Sally Ride broke barriers as the first woman in space, NASA scientists estimated that she would need 100 tampons for one week in space (1). While this is an overestimation of the number of tampons the average individual requires for a period, the true cost of menstrual products is a significant financial burden for many individuals who menstruate. The rising costs of menstrual products are also accompanied by a lack of general knowledge, stigmas, negative connotations, and shameful sentiments concerning menstruation (2).

Menstruation exerts an impact beyond reproductive health, from mental health to gender inequality and economic disparity, even to the prevalence of serious diseases such as cervical cancer. Strong stigmas and lack of education about period products may impact the choice of products, creating an opportunity for pharmacists to contribute to increasing access to menstrual products (3).

## 2 Impact of periods on daily life

### 2.1 Period poverty

Period poverty occurs when someone lacks access to menstrual products, education, sanitation, and other hygiene resources (i.e., laundry and clean water), which are all needed during a period (4). This is a prevalent yet rarely discussed issue facing those who menstruate. A recent study of 471 United States (U.S.) college students found that one-quarter of students reported period poverty in the past year, including 10% for whom it was a monthly occurrence (4). Among U.S. women on low incomes 64% reported period poverty during the previous year, a monthly occurrence for one-fifth of respondents (5). Lastly, another recent study indicated that the COVID-19 pandemic made it increasingly difficult to access period products. The cross-sectional study of a representative sample of U.S. adults found that 29% of participants struggled to purchase period products in the past year, and 18% missed work due to a lack of period products (6).

To estimate the costs of period products, we collected pricing data from four major retailers (Table 1). For each product, the average recommended duration of use per manufacturer was used to calculate the average cost of each product per period. The average duration and frequency of periods were used to calculate the average cost of each product per period, year, and lifetime. The average period is 3–7 days, so an average of 5 days per period was used (7). The average individual who menstruates has a period every 28 days and menstruates for 38 years, equating to around 456 periods per lifetime or 2,280 days spent menstruating (8). For more detailed information, see the Supplementary information.

**Table 1:** Menstrual products comparison.

Consideration	Products				
	Pads	Tampons	Discs	Cups	Underwear
	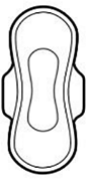	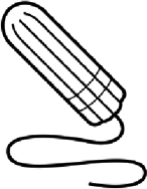	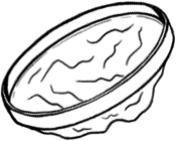	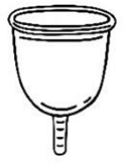	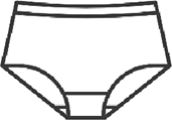
Materials	Polypropylene/polyethylene fibers	Rayon, cotton, or mixture	Silicone, polymers, or combination	Medical-grade silicone, rubber, latex, or elastomer	Microfiber polyester, PUL, PFAS
Usability	Disposable	Disposable	Disposable/ reusable	Reusable	Disposable/reusable
Average cost per year^a^	$64	$71	$158/$29	$28	$178/$21
Average cost per lifetime^b^	$2,420	$2,684	$5,990/$232	$228	$6,772/$802
Recommended duration of use	4–8 h	4–8 h	Up to 12 h	Up to 12 h	Up to 12 h
Safety notes	Absorbs and stores menstrual fluid away from the body. Generally safe when used as recommended. Low risk of TSS	Absorbs and stores menstrual fluid inside vagina. Generally safe when used as recommended. Higher risk of TSS with prolonged use	Stores menstrual fluid inside vagina; rarely reported to cause TSS	Stores menstrual fluid inside vagina; rarely reported to cause TSS if properly sterilized between uses	Absorbs and stores menstrual fluid away from the body. Advertised to have no risk of TSS, but insufficient evidence to support this

PUL, Polyurethane Laminate; PFAS, per- and polyfluoroalkyl substances; TSS, toxic shock syndrome.

^a^ Estimated based on average of 5 days per period and 12 periods per year.

^b^ Estimated based on average of 456 periods per lifetime.

Additionally, menstruation may incur other indirect costs; for example, if someone experiences a heavy flow, it may necessitate the purchase of new underwear, linens, or clothes. Some individuals may also need to purchase medications such as ibuprofen, heating pads, or even transcutaneous electrical nerve stimulation (TENS) to alleviate the pain accompanying menstruation. Furthermore, individuals who menstruate may need a combination of products throughout their period. Individuals who prefer tampons may switch to overnight pads for sleeping and panty liners on their lighter days. Despite the necessity of period products, they are not covered by food stamps and subsidies under the Women, Infants, and Children (WIC) and Supplemental Nutritional Assistance Program (SNAP) (9, 10).

### 2.2 Quality of life

Period poverty transcends financial concerns. For those experiencing period poverty, their lack of access to menstrual products can negatively impact health, education, physical activity, and even the ability to work A study of nearly 33,000 female respondents found that 13.8% of women experienced absenteeism during their menses, and 80.7% reported presenteeism (showing up for work without being productive) during menstrual symptoms (11). In 2023, Spain became the first country in the world to require paid menstrual leave to accommodate individuals with incapacitating menstruation (12).

A study of high school students found that two-thirds (66.9%) of students surveyed reported using at least one of the school's resources to obtain period products and one-third of participants (33.6%) missed school due to lacking period products (13). Another study of students in the UK found that one in ten girls aged 14–21 can't regularly afford menstrual products and are forced to choose between staying home or using makeshift products such as paper or socks (14). An alarming 80% of adolescent girls also experienced uncomfortable menstrual symptoms such as heavy or irregular bleeding, but 27% of respondents were too embarrassed to discuss their symptoms with their healthcare providers. Feelings of shame, weakness, and embarrassment outweigh their inclination to seek medical support for these concerning symptoms. These feelings are a foundation for increased emotional anxiety with numerous potential effects. It is also notable that people facing poverty have higher rates of depression (4). A 2021 study of women in college found that 68.1% of those facing period poverty showed symptoms of moderate-to-severe depression.

### 2.3 Physical activity limitations

The many benefits of physical exercise are well-known, yet menstruation accompanied by acute or chronic pain may impose temporary limitations on physical activity (15). The physical pain that some women experience with dysmenorrhea can be substantially disabling. Several randomized, placebo-controlled studies have shown that non-steroidal anti-inflammatory drugs (NSAIDs), like ibuprofen, can provide efficient pain relief. Nonetheless, the stigma and embarrassment associated with discussing menstruation with healthcare providers may preclude individuals suffering from dysmenorrhea from seeking medical attention for their pain. NSAIDs also have a risk for gastrointestinal adverse events when being used chronically.

During the menstrual cycle, athletes undergo added stress as the stigma of menstruation and lack of acceptance discourages them and affects focus, confidence, performance, and mental health. A BBC Elite British Sportswomen's Survey found that 60% of athlete respondents said their performance had been affected by their period, and they had missed training or competitions due to their period (16). However, 40% said they did not feel comfortable discussing their period with coaches, and many took the contraceptive pill to control their menstrual cycle.

## 3 Availability and safety of period products

Menstrual hygiene products can be classified as internal (e.g., tampons, cups, and discs) or external products (e.g., pads and underwear) (7). Reusable products (e.g., cups, discs, and underwear) have been gaining attention due to their environmentally friendly and cost-saving implications (7, 17). A study found that although about 81.8% of women considered menstrual hygiene products harmful, only 43.6% of women had read the instructions on their choice of products or inquired (30.7%) about their composition (7).

### 3.1 Pads

Disposable menstrual pads (also known as sanitary pads) were the first commercially popular menstrual product in the United States (18). They are generally comprise three layers; polypropylene/polyethylene fibers for absorption, an emollient for contact with the skin, and an adhesive layer to affix them to underwear. Menstrual pads are designed to store menstrual fluid away from the body. The CDC recommends changing menstrual pads every few hours, which can vary based on the size, brand, and menstrual flow of the user (19).

Menstrual pads generally have a good safety profile. A risk of using menstrual pads is the unintended absorption of chemicals. The mucous membranes of the vagina and vulva rapidly absorb chemicals without metabolizing them, making the vaginal route ideal for drug delivery. One study found that vaginal application of estradiol resulted in blood serum levels 10 times higher than those following oral dosing (20). Some women may be unknowingly exposed to allergens, especially in scented menstrual pads (21). Studies have mainly shown that pads are not associated with any significant gynecological, dermatological, or microbiological effects (22).

### 3.2 Tampons

Tampons generally comprise a cellulose absorbent material with either rayon, cotton, or a mixture (23). Some tampons may also have fragrances or dyes added for deodorizing and aesthetic purposes, respectively. They are disposable products with a recommended duration of use of 4–8 h; as with pads, this time varies based on the size, manufacturer, and menstrual flow of the user (19).

One of the most recognized risks associated with tampons is toxic shock syndrome (TSS). Studies performed in the 1980s associating tampon use with TSS led to the inclusion of TSS warning labels on tampon boxes and uniform standards for absorbency labeling (24). Toxins produced by concentrated *Staphylococcus aureus* in the presence of synthetic fibers can lead to a potentially deadly toxin-mediated disease. While previous studies have proposed that tampons containing rayon have a higher risk of TSS, this has been disproven. The Centers for Disease Control and Prevention (CDC) recommendation states that individuals should use tampons of the lowest absorbency to control menstrual flow to reduce the risk of TSS (19).

### 3.3 Cups

Menstrual cups generally comprise silicone, thermoplastic elastomers (TPEs), or natural rubber (25). They are designed to be worn for up to 12 h, cleaned, and then reused. Like pads, the size, shape, and material of menstrual cups may vary. Selection of the correct size and shape is instrumental to the level of comfort and success someone will experience when using menstrual cups. Different folding strategies exist to ensure proper placement in the vagina, and users can verify correct placement by checking for folds after placement or tugging on the stem to ensure the cup remains in place (26).

A systematic review examining 43 studies on the safety of menstrual cups concluded that these are a safe method of managing periods (27). Moreover, the study found no changes in the vaginal flora or increased risk of infection. Some risk of TSS still remains with menstrual cups, but TSS risk labeling is not required. A unique safety factor concerning menstrual cups is the risk of device impaction requiring medical removal. Some people may have difficulty removing the cup or require professional assistance (27).

### 3.4 Discs

Menstrual discs resemble cups but are generally disposable, although reusable discs are also available. Discs have a rubber or silicone rim and an expandable plastic or silicone collection basin that is wider but shallower than cups. The flexibility of the discs contributes to an increased level of comfort for some users; however, this flexibility can also increase the risk of leaks. It is recommended that these devices be used for a maximum of 12 h. Discs share a similar safety profile to cups, with a relatively smaller risk of causing TSS when used appropriately (28).

### 3.5 Underwear

Menstrual underwear is the newest product to be introduced to the market. It is available as reusable or disposable products resembling normal underwear with built in absorbency. It can be worn alone or combined with other menstrual products for an added layer of protection. Menstrual underwear has seen an increase in use as a growing number of menstruating people, particularly Gen Z consumers, are proactively seeking safer, more environmentally friendly products (29).

Perceptions of period underwear may be worsened by recent settlements regarding the presence of short-chain per- and polyfluoroalkyl substances (PFASs) in these products (29). PFASs can potentially cause a wide variety of negative health effects in humans (30). A lawsuit alleged that Thinx misled consumers, marketing itself as an organic, sustainable, and nontoxic alternative to traditional one-use menstrual products, including pads and tampons. Knix, a similar company based out of Canada, also reached a settlement over the presence of PFAS in their products despite claiming to be PFAS free (31). Due to the novelty of period underwear, limited studies are available on their long-term safety.

## 4 Pharmacists' role in increasing access to menstrual products

### 4.1 Education

A pharmacist's principal role in increasing access to period products is through education. Sex education in schools typically fails to incorporate education about the various menstrual products available (17). This leaves individuals requiring menstruation products hesitant to try new products. A United Kingdom study found that teachers perceived a need for better menstrual education to improve health and school performance. Around 80% of the teachers felt they would be better prepared to provide education if they were given appropriate training. Trant et al. found that only 44% of pediatric providers ask patients about menstrual products (32). The stigma surrounding menstrual health could be a barrier to these conversations.

Pharmacists have been critical to building the trust necessary to reduce vaccine hesitancy by leveraging their trusted relationships with the community (33). The same relational communication can be used to quell hesitation about different menstrual products. Several products are available for menstruation, ranging from disposable pads/tampons to reusable cups (Table 1). Most menstrual products are purchased from pharmacies and pharmacists are the most accessible healthcare professional, providing ample education opportunities. Therefore, pharmacists should be familiar with the menstrual products available in their pharmacy, including their advantages and disadvantages. The FDA regulates most menstrual products, and pharmacists should be aware of the proper use, storage, and safety precautions for these devices. Pharmacists can then guide patients in making cost-effective and health-conscious decisions based on their patients' individual needs.

A study by Moon et al. showed that even women who are comfortable using only one type of menstrual product are interested in and desire to try other menstrual products (34). Their curiosity is hindered by their lack of knowledge or experience with the other products. The study also showed that providing education on menstrual products other than pads positively changed study perspectives on other menstrual products. Pharmacists can use their relationships and expertise to increase patient willingness to try products such as reusable cups and discs. These alternatives to pads and tampons could provide extensive cost savings over the lifetime of a patient.

### 4.2 Legislation

**Figure 1:**
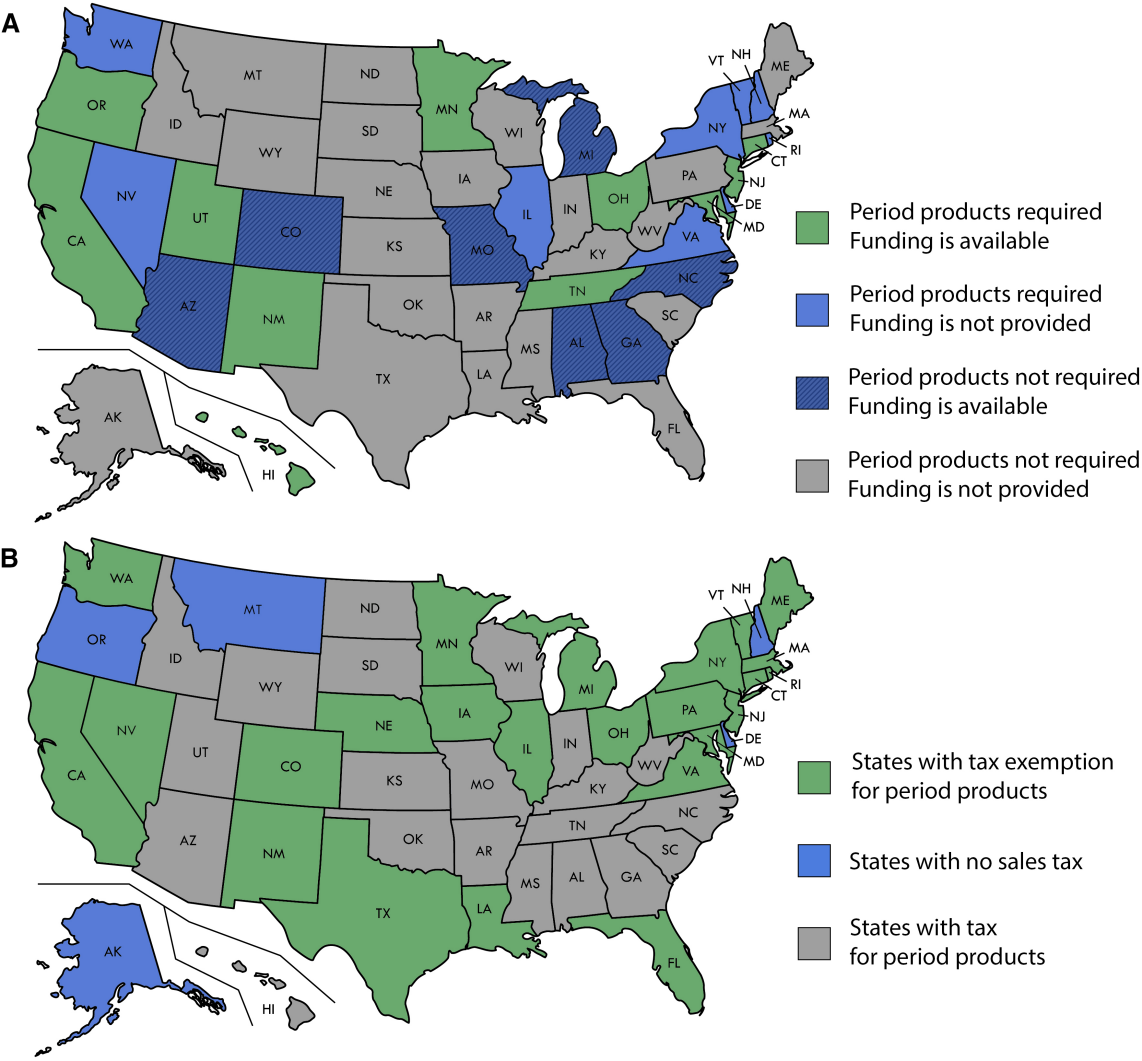
(**A**) State policy on the requirement of period products in schools. (**B**) State policy on period products tax.

Pharmacists can also increase access to menstrual products through advocacy and legislation. In 2018, New York was the first state to pass a law requiring public schools with grades 6–12 to provide free menstrual products in restrooms (35). As of June 2023, 20 states addressed access to menstrual products in schools. The policies vary from state to state, as does the funding to execute them (Figure 1A). Some states annually appropriate money to provide the products or offer reimbursement for a portion of the cost. Other states require schools to provide the products but do not provide additional funding. The location of the products also varies, with some states only requiring them to be present in female bathrooms while others stipulate that they also be available in male and unisex bathrooms, providing inclusivity for transgender and nonbinary students.

Pharmacists should champion inclusive policies that directly increase access to such menstrual products at the state and national levels. Scotland set an international precedence in 2020 becoming the first country to provide free tampons and pads to anyone requiring them (36). The products are available in community centers, youth clubs, and community pharmacies. This highlights the potential for pharmacies to function as safe places for individuals requiring access or education on menstrual products.

Other potential policy changes to advocate for are designating menstrual products as necessities to make them tax-exempt. While menstrual products are considered medical devices, they cannot be purchased through government funding assistance. Unlike products such as groceries, prescriptions, and prosthetics, menstrual products are considered tangible individual property, which incurs sales tax. If menstrual products were classified as non-luxury necessities, they would be tax-exempt. Currently, 24 states deem menstrual products fully exempt from tax (Figure 1B) (37). Those who menstruate face higher prices on menstrual products and other gender-specific products and services marketed as “for women,” also known as the pink tax. This results in high prices for menstrual products and the resulting sales tax, also known as the tampon tax, charged on feminine hygiene products, while other products deemed necessities are granted sales tax exemption.

### 4.3 Insurance reimbursements

Finally, pharmacists can educate patients on the potential coverage of menstrual products through their health insurance. In 2022, Congress passed the Coronavirus Aid, Relief, and Economic Security (CARES) Act, which made certain menstrual products reimbursable through flexible spending accounts (FSAs) or health savings accounts (HSAs) (38). Some retailers advertise which products are FSA-eligible, helping people identify which products will be most cost-effective for them.

HSAs offer patients with high deductible insurance plans an opportunity to save for healthcare expenses while saving on their taxes. A 2020 survey revealed that an estimated 32.5% of adults had no HSA, and 9.1% did not know whether they had an HSA (39). A significant amount of attention has been devoted to increasing access to HSAs and leveraging them to decrease overall healthcare costs. Between 2019 and 2021, 23 bills were introduced to expand eligibility for HSAs. HSAs were also incorporated into the presidential federal budgets in 2020 and 2021. Many patients may be unaware that their health insurance offers these accounts or may not be versed in how to use them. Pharmacists can encourage patients to explore offers included in their healthcare plans and maximize their healthcare benefits.

## 5 Discussion

The financial burden menstrual products impose on individuals requiring them is often underestimated. Many Americans struggle with period poverty; however, pharmacists can have a multifaceted role in increasing access to period products through education and advocacy.

Our findings are constrained by a potential underestimation of the total cost of menstruation. The estimates provided were derived from only four major retailers. We also did not account for varying tax rates on period products or inflation. In addition, we did not factor in the varying combination of products individuals may use during menstruation or account for changing products more frequently than recommended, which would greatly increase the number of products used per cycle. Lastly, we did not factor in the costs outside of period products (e.g., pain medications, lost labor, accessories). Despite these constraints, it is evident that reusable menstrual products are the most cost-effective over time.

Pharmacists can also advocate for inclusive policies, such as ensuring free access to menstrual products in schools and reclassifying products as tax-exempt necessities. Finally, pharmacists can educate patients about insurance reimbursement options, including leveraging FSAs and HSAs to alleviate the financial burdens associated with menstruation. Future studies can elucidate the true cost-savings of implementing these strategies.

## 6 Conclusion

Menstruation is a key component of reproductive health, and pharmacists are underutilized in public health initiatives to provide education and improve access to these necessary products. Pharmacists are easily accessible healthcare professionals who can provide education on menstrual products, highlight cost-saving practices, and champion legislation that promotes increasing access to menstrual products. Through efforts rooted in education and advocacy, pharmacists can improve the quality of life of individuals who menstruate.

## Data Availability

The original contributions presented in the study are included in the article/Supplementary Material, further inquiries can be directed to the corresponding author.

## Supplementary material

Supplementary Data Sheet 1 Supplementary materials include the aggregation data analyzed to determine the average cost of period products as summarized in Table 1, as well as individual state level information on the status of taxes on period products and school requirements for period products as summarized in Figure 1.
